# Effect of hetero-atom doping on the electrocatalytic properties of graphene quantum dots for oxygen reduction reaction

**DOI:** 10.1038/s41598-023-31854-8

**Published:** 2023-03-30

**Authors:** Mrigaraj Goswami, Sneha Mandal, Vijayamohanan K. Pillai

**Affiliations:** Department of Chemistry, Indian Institute of Science Education and Research (IISER), Karakambadi Road, Mangalam, Tirupati, 517507 India

**Keywords:** Chemistry, Energy science and technology, Engineering, Materials science, Nanoscience and technology

## Abstract

Oxygen reduction is an important reaction involved in a diverse variety of energy storage devices and also in many chemical and biological processes. However, the high cost of suitable catalysts like platinum, rhodium, and iridium proves to be a major obstacle for its commercialization. Consequently, many new materials have emerged in recent years such as various forms of carbon, carbides, nitrides, core–shell particles, Mxenes, and transition metal complexes as alternatives to platinum and other noble metals for oxygen reduction reaction (ORR). Among these, Graphene Quantum Dots (GQDs) as metal-free alternatives have captured universal attention, since electrocatalytic properties can be tuned not only by size and functionalization but by heteroatom doping also. We discuss electrocatalytic properties of GQDs (approximate size 3–5 nm) with specific dopants such as N and S focusing on their synergistic effects of co-doping, prepared by solvothermal routes. Cyclic Voltammetry shows benefits of doping as lowering of the onset potentials while steady-state Galvanostatic Tafel polarization measurements show a clear difference in the apparent Tafel slope, along with enhanced exchange current densities, suggesting higher rate constants.

## Introduction

Oxygen reduction reaction (ORR) plays a vital role in metal-air batteries, fuel cells, corrosion^1^ and in many biological systems^2^. Platinum is considered to be the best electrocatalyst for ORR, but its limited availability and higher cost render large-scale commercialization difficult. For example, in commercial fuel cell stacks, Pt is often responsible for 50% of the total cost^3^. Consequently, several research groups are working to replace Pt with specially designed metal-free catalysts or with core–shell systems, having a thin layer of Pt as the shell on an abundant core like Fe or Sn^4^.

Carbon being an environment-friendly and abundant material, has the potential to be a suitable metal-free electrocatalyst for ORR and many groups have carried out extensive studies in recent times^5^. However, there are still some challenges with carbon-based electrocatalysts, like functionalization, carbon corrosion in the fuel cell environment, and its long-term stability, preventing wide-scale applications. Graphene Quantum Dots (GQDs), are quasi-zero-dimensional crystalline nanostructures generated conceptually when we stack a few layers of Graphene on top of each other. Their lateral sizes may be larger (even 80–100 nm), but the finite number of layers (approximately 3–8 nm) and edge states control their electronic behaviour^6^. They exhibit unique properties like quantum confinement, fluorescence, tuneable band-gap, highly exposed surface and edge effects, and low cytotoxicity, which make them suitable to deploy for applications in various fields like luminescence^7^, catalysis^8^, and biology^9^. Over the past decade, GQDs have attracted considerable attention as an efficient alternative to Pt for ORR. Factors like size, doping, surface functionalization and regulation of the number of available active sites in GQDs play an important role in controlling the efficiency of ORR. For example, Dai et al. used nitrogen-doped GQDs as catalysts for ORR, revealing better performance compared to the N-free counterparts, along with enhanced luminescence^10^. Like nitrogen, sulfur can also be used as a dopant to enhance the electrocatalytic performance of GQDs. Further, Baek et al. doped GQDs with sulfur in order to accomplish better ORR performance using the peak current density and the onset potential^11^. Nitrogen and sulfur can also be co-doped on GQDs to enhance the ORR. For instance, Liang et al. used N,S co-doped Graphene as catalysts for ORR, comparing its performance against N-doped Graphene, S-doped Graphene and pristine Graphene. Co-doping shows better performance than single atom doping, exchange current densities being the experimental evidence^12^. Size also plays a critical role in the catalytic performances of GQDs, as seen in the case of N-doped GQDs, catalytic performance is inversely proportional to the size, based on the onset potentials and the peak current densities^13^. However, debate is still going on with respect to the contribution of the site of nitrogen doping and their role, as some groups suggest that the graphitic nitrogen is responsible for ORR^14^, whereas others believe that it is Pyridinic nitrogen^15^. Recent DFT calculations show that Pyridinic nitrogen at the armchair edges demonstrates the highest activity for ORR under acidic conditions^16,17^. Also, in sulfur doped and nitrogen-sulfur co-doped GQDs, carbon–sulfur bond formation induces charge and spin density, enhancing the ORR performance^17,18^. Despite these recent studies, some fundamental questions related to ORR performance with respect to doping and co-doping still remain elusive. Also, many of the alternate materials lack sustained stability and more studies are hence needed to explore their potential applications.

Herein we report a comparative study of N-doped, S-doped, and N,S co-doped GQDs for electrocatalytic ORR activity in alkaline medium. The physical characteristics of the GQDs are investigated using Powder X-ray Diffraction (XRD), UV–Vis, Fluorescence, and FTIR spectroscopy, while the electrocatalytic properties are explored using Cyclic Voltammetry, and Galvanostatic, steady-state, Tafel polarization measurements to clearly indicate an order of magnitude change in kinetic parameters with heteroatom doping. These results suggest their potential utility as metal-free electrocatalysts to replace noble metals currently used in fuel cells and metal-air batteries in order to have lower costs and better efficiency.

## Experimental

Pristine GQDs (GQD-MW) were synthesized by microwave-assisted acidic exfoliation of Graphene Oxide (GO) (Supplementary Information S-1). N-doped (N-GQDs), S-doped(S-GQDs) and N,S co-doped GQDs (N,S GQDs)were synthesized using a solvothermal route. (S-2).

UV–Vis measurements were carried out in an Agilent Cary UV instrument, at room temperature in water while fluorescence emission data were taken in a Jasco FP 8500 fluorimeter, with a Xenon lamp as the light source. Like the UV–Vis measurements, the emission spectra of N-GQDs and N,S-GQDs in water, and S-GQDs in N-Methyl Pyrrolidone (NMP), were taken because of solubility restrictions. The FTIR spectra of the GQDs were collected using a Perkin-Elmer ATR instrument.

Electrochemical properties were determined using a 3-electrode setup (Biologic SP200 electrochemical workstation) using a Glassy carbon electrode coated with 40 µl of the quantum dot suspension (1 mg/ml) as the working, a Pt coil as the counter, and a pre-calibrated Hg/HgO electrode as the reference electrode respectively. In order to rule out the possibility of platinum contamination, separate experiments were carried out using graphite counter electrodes. All measurements were taken in basic conditions (0.1 M KOH) saturated with oxygen, while blank measurements were carried out in 0.1 M KOH purged with argon. Cyclic voltammograms were taken in a restricted potential window (from − 0.8 V to 0.4 V) at different scan rates based on preliminary voltametric measurements. In order to calculate exchange current densities and apparent Tafel slopes, Galvanostatic steady-state polarization measurements were taken in 0.1 M KOH purged continuously with O_2_.

### Ethics approval and consent to participate

The work done is original, complete, and has not been submitted elsewhere for publication.

## Results and discussion

**Figure 1:**
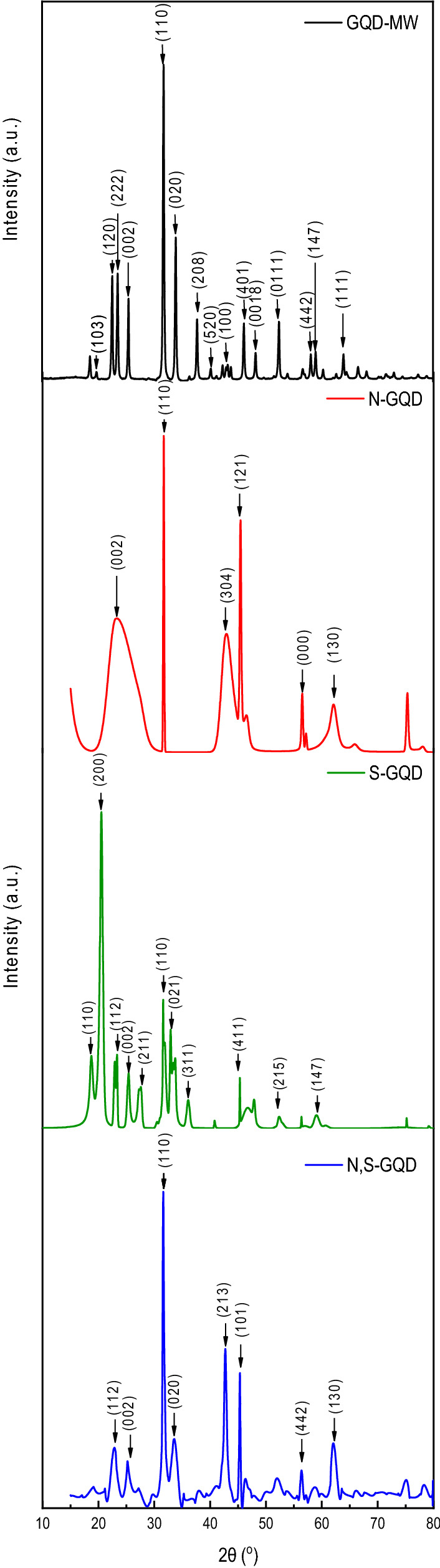
Comparison of the Powder XRD patterns of Pristine, S-doped, N-doped, and N,S co-doped GQDs. XRD patterns were taken from 15 to 80° at a scan rate of 1° per minute.

Figure 1 shows a comparison of the Powder XRD profiles of the pristine and doped GQDs. Doping of GQDs is expected to cause lattice expansion or contraction, depending upon the size of the dopant atom. As we see in Fig. 1, there are clear shifts in (002) and (110) peaks in all three doped systems, suggesting changes in the lattice parameters^10^. Atomic radii of nitrogen atoms and sulfur atoms are 56 pm and 100 pm respectively, so incorporating them into GQD lattice leads to contraction^19,20^ and expansion respectively^21,22^. The d_(002)_ spacing values come out to be around 3.4 A^0^, and the d_(110)_ is around 2.8 A^0^ for all the GQDs respectively, which are in excellent agreement with other reports as well^19–22^.

**Figure 2:**
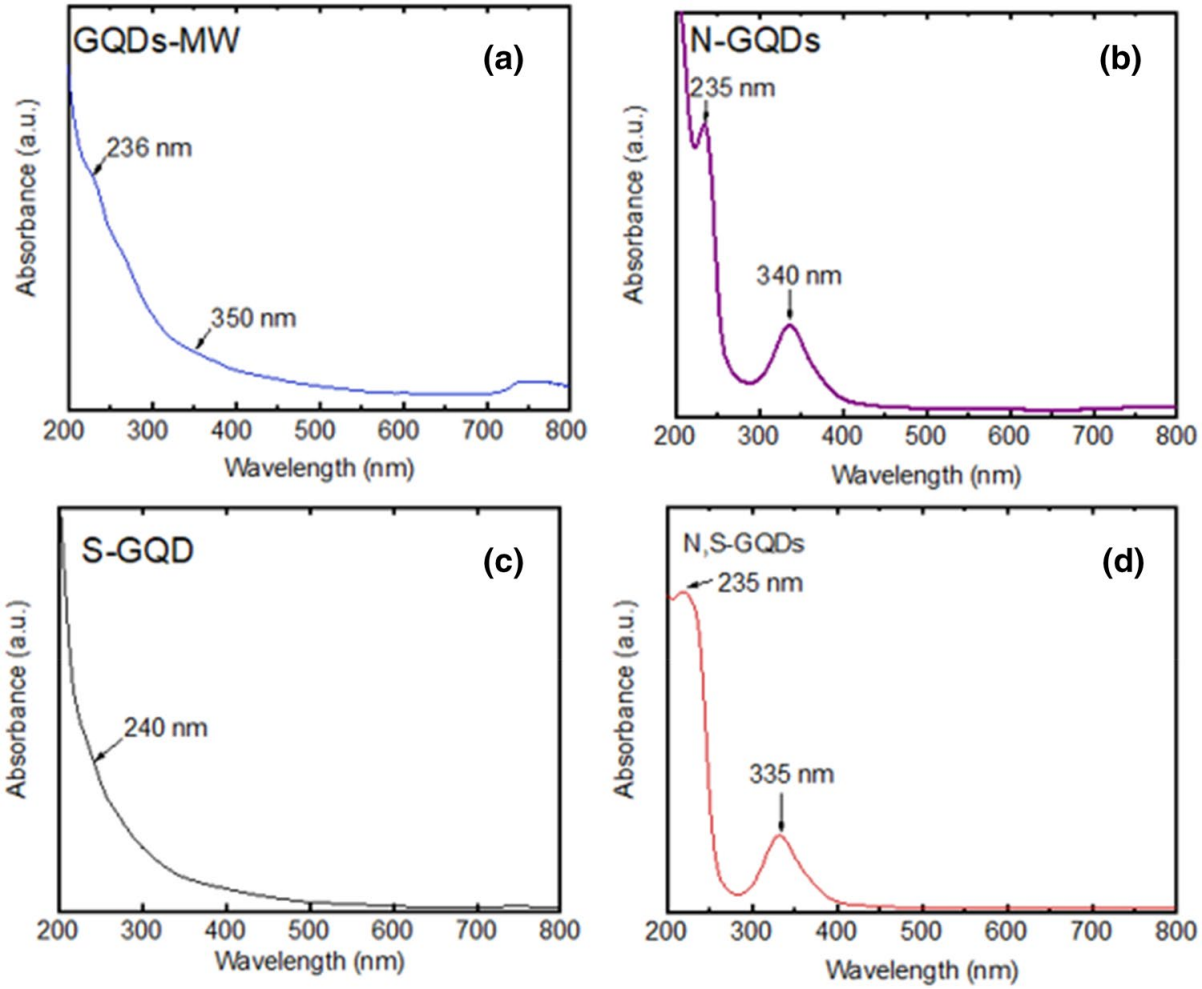
UV–Vis spectra of (**a**) Pristine GQDs, (**b**) N-GQDs, (**c**) S-GQDs and (**d**) N,S-GQDs. Measurements were taken from 200 to 800 nm at room temperature.

Figure 2 shows the UV–Vis spectra of the GQDs where one peak at 236 nm, corresponding to the π to π* transition of C=C bonds, and another at 350 nm, corresponding to the n to π* transition in the C=O bonds^23,24^ in the pristine GQDs^25^ are seen. In the doped GQDs, we observe one peak at 235 nm for N-doped GQDs, 240 nm for S-doped GQDs and at 235 nm for the N,S co-doped GQDs, corresponding to π to π^*^ transitions^26–28^, and another at 350 nm, 340 nm for the pristine and N-doped respectively corresponding to n to π^*^ transition^26,29^. Interestingly, in the S-doped GQDs, no peak at 340 nm is observed, probably due to the lesser number of C=O bonds, and also because of its symmetry forbidden nature^30^. In the N and N,S co-doped GQDs, the peak at around 340 nm is relatively intense compared to that of the pristine GQDs, perhaps due to a decrease in the oscillator strength of n to π* transition^31^.

**Figure 3:**
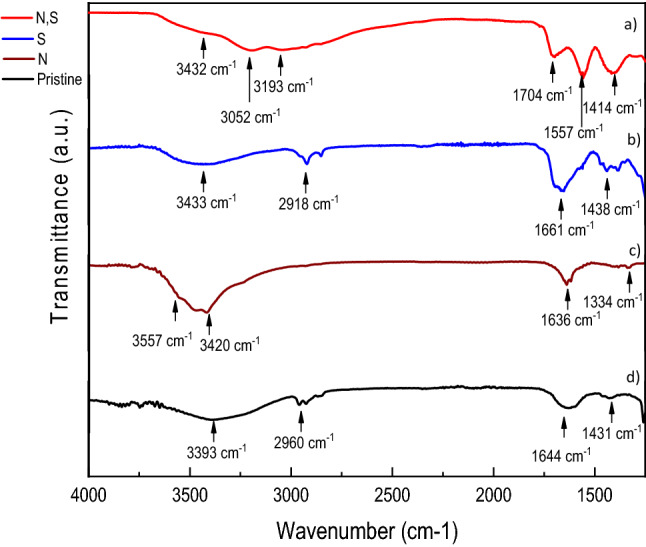
A Comparison of the FTIR spectra of (**a**) N,S-GQDs, (**b**) S-GQDs, (**c**) N-GQDs and (**d**) Pristine GQDs.

Figure 3 compares the FTIR spectra of the GQDs. We can observe –OH peaks in all the GQDs, in the neighbourhood of 3400 cm^−1^ ^29^. A doublet corresponding to –C–H stretch is also observed in GQDs at around 2900–2960 cm^−1^ while –N–H stretch is observed at around 3400 cm^−1^ ^32^. C=C stretch is also observed. In the S-doped and N,S co-doped GQDs, we can see a sulfonyl stretching at around 1438 and 1414 cm ^−1^ respectively. In the range of 1630–1710 cm^−1^, we can see a peak corresponding to conjugated ketonic C=O stretching, which shows across different GQDs, probably caused by the introduction of dopants into the conjugated system^33^. In earlier work done by our group on synthesizing N,S co-doped GQDs, XPS studies showed the presence of C, O, N and S with atomic concentrations of 68%, 9.6%, 11.6% 8.2% and 0.1% respectively, which is in agreement with our EDX data. The composition of the GQDs could be understood well in terms of heteroatom doping, and it is well known that different N doped species present in pyridinic and graphitic moiety produce different catalytically active sites. For example, S 2p spectrum has two peaks centred at 164.7 and 169.1 eV respectively indicating the presence of S in two forms while four kinds of N bonding could be seen from 398.16 eV (owing to pyridinic), 399.62 eV (pyrrolic), 400.73 eV (quartenary) and 401.85 (quarternary valley)REF^19^.

**Figure 4:**
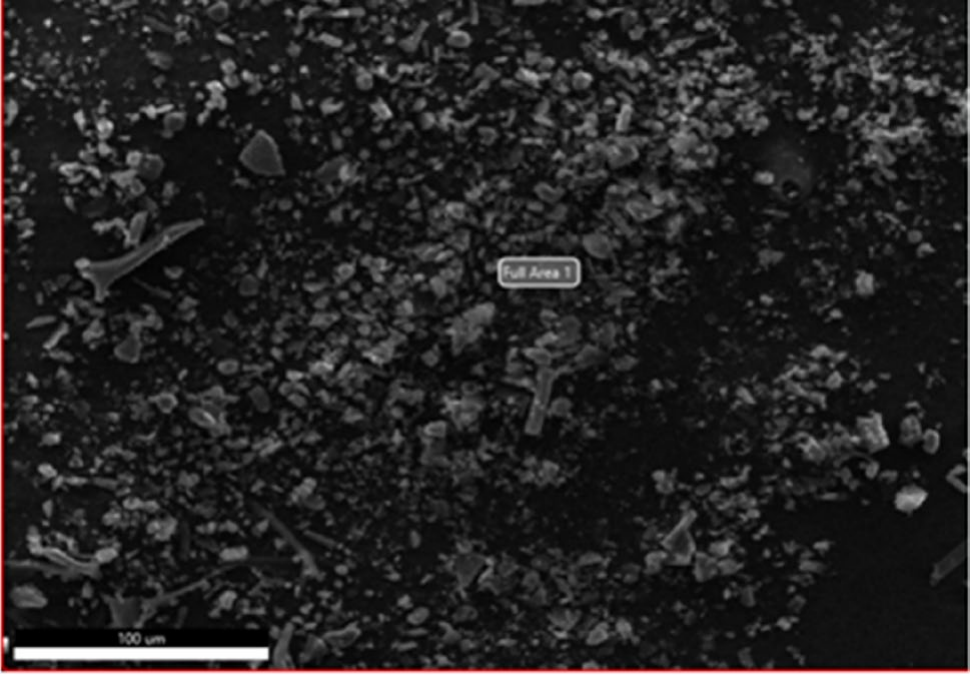
representative SEM of the N,S co-doped GQDs and the EDX data indicated elemental composition as 55.6% of C, 7.0% of N, 37.3 of O, and 0.1% of S by atomic weight percentage.

Figure 4 shows the surface morphology as evidenced by the Scanning Electron Micrograph of the N,S co-doped GQDs. The GQDs appear to be granular with more or less uniform particles with no visible signs of agglomeration. The EDX data reveal 55.6% of C, 7.0% of N, 37.3 of O, and 0.1% of S by atomic weight percentage in the sample, showing that the GQDs has been successfully doped with both N and S. However, morphologies of GQDs co-doped with N and S were very similar to that of original GQDs as supported by the, microstructural analysis since structural, topological or edge defects could not be resolved among all these samples.

**Figure 5:**
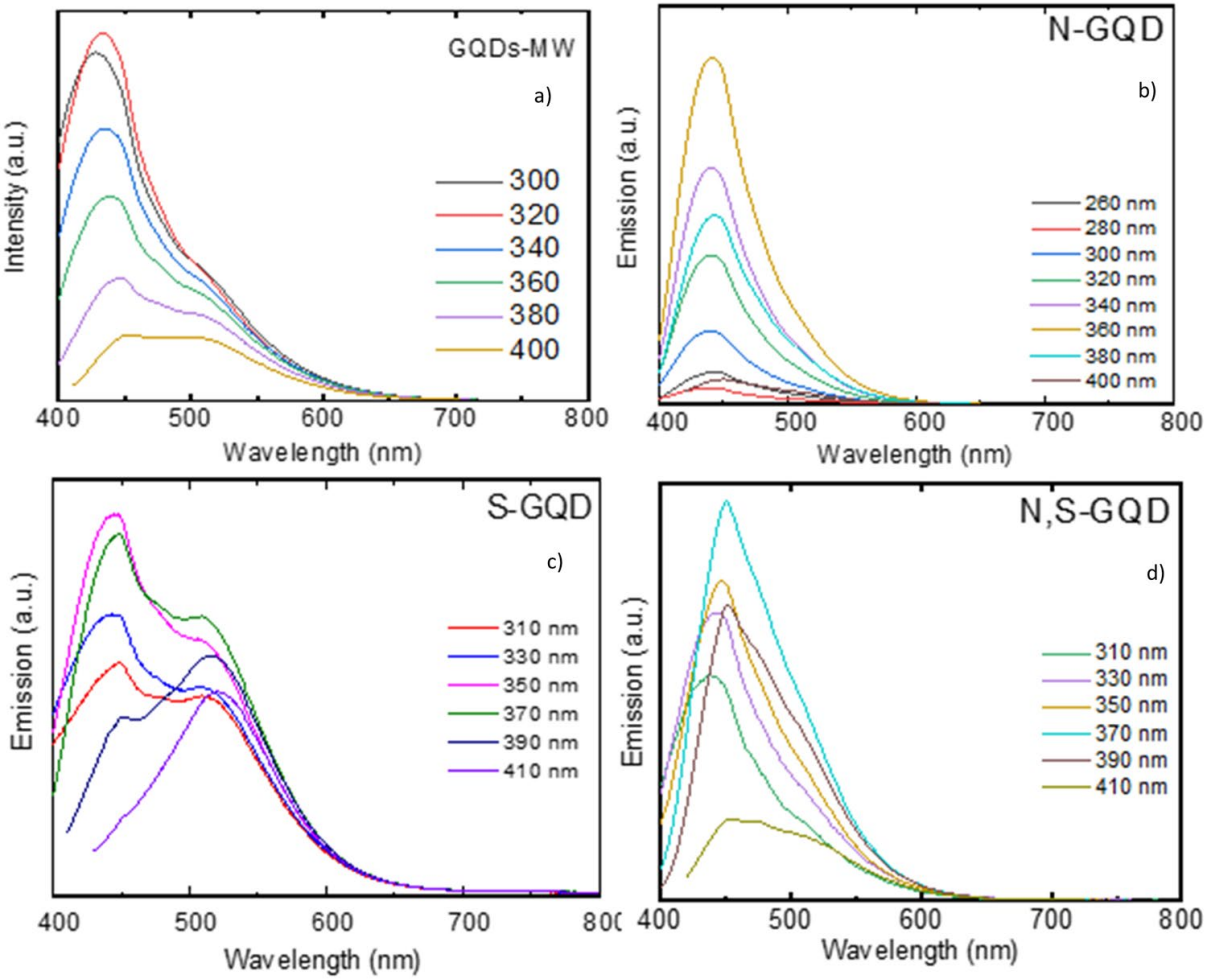
Emission spectra of (**a**) GQDs-MW, (**b**) N-GQDs, (**c**) S-GQDs and (**d**) N,S GQDs, showing that the approximate size of the GQDs fall in the range of 2–5 nm.

Fluorescence emission spectra of the GQDs taken using an excitation wavelength of 300 to 410 nm-are shown in Fig. 5. In the pristine GQDs, we get a peak at 450 nm in the emission spectrum. Under UV excitation, all the GQDs exhibit blue-green luminescence. We see an excitation-independent luminescence profile in the N-doped GQDs, with the excitation maxima occurring at 450 nm. In the N,S co-doped GQDs, we can observe the excitation maxima to be at around 450 nm, but unlike the case of N-doped GQDs, it is excitation dependent. This can be attributed to the various surface states present on the N,S-GQDs^33^. In the fluorescence spectrum of S-doped GQDs, we can observe dual emission, one peak occurring at around 450 nm, and the other peak at 520 nm respectively. The peak at 450 nm shows excitation independence (inter-band transition), whereas the peak at 520 nm is excitation dependent, perhaps due to the presence of surface groups^33^. These surface groups are also believed to be responsible for the lack of sustained stability especially in dry conditions when the doped GQDs are stored more than few weeks.

**Figure 6:**
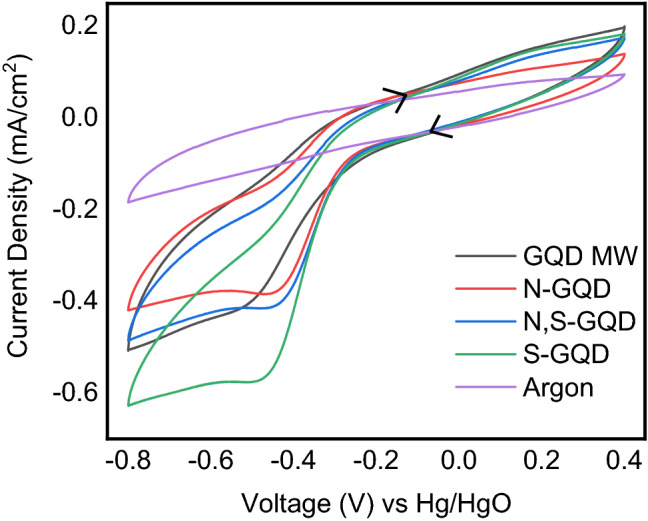
Comparison of Cyclic Voltammograms in O_2_ saturated 0.1 M KOH at 100 mV/s in the potential window of − 0.8 to 0.4 V; working electrode: Glassy Carbon, Counter electrode: Pt coil, Reference electrode: Hg/HgO, the fifth cycle is shown for all the GQDs. A blank (base line) was taken with Argon purged KOH to make sure no impurities are there while doing the measurements.

**Figure7:**
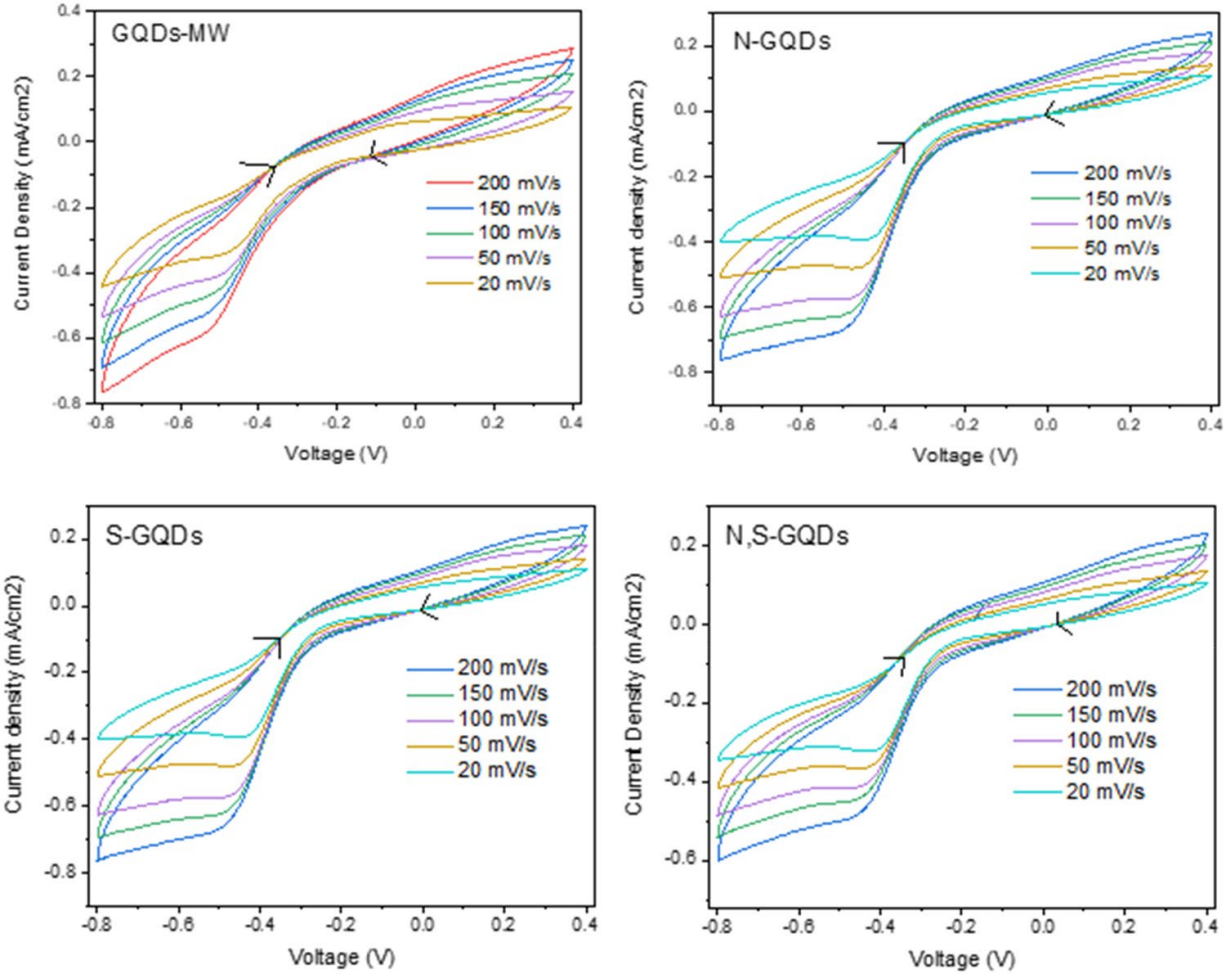
Scan Rate dependent CVs of the GQDs in O_2_ saturated 0.1 M KOH. Scan rates were changed from 200 to 20 mV/s, taken from − 0.8 to 0.4 V.

Figure 6 depicts superimposed Cyclic Voltammograms of all the GQDs at a constant scan rate of 100 mV/s. In the pristine GQDs, we observe a peak at − 0.5 V while for the case of doped GQDs, the peak shifts to − 0.45, − 0.48 and − 0.47 V for N-doped, S-doped and N,S co-doped GQDs respectively. This shows that Oxygen reduction is thermodynamically more favourable in the case of doped GQDs compared to the pristine GQDs. The observed OCV values (210 mV for the pristine, 230 mV, 300 mV and 250 mV for the N-doped, S-doped, and N,S co-doped GQDs) also indicate this order despite more variability (≅ 5 mV) . The current density at a typical voltage like − 0.45 V (approximately 650 mV overpotential) is 0.34 mA/cm^2^ in the pristine GQDs, 0.38 mA/cm^2^ in the N-doped GQDs, 0.545 mA/cm^2^ in the S-doped GQDs, and 0.411 mA/cm^2^ in the case of N,S co-doped GQDs. This is also in excellent agreement with RDE studies on similar GQDs by other groups^31^. Also, from our earlier RDE studies on the ORR performance of N-doped GQDs, we found out that 4 electron pathway is preferred in the case of N-doped GQDs in basic condition^13^. This is further confirmed by the scan rate dependence of the voltammograms indicated in Fig. 7.

It is well known that Oxygen Reduction in alkaline media can undergo via a two-electron pathway, or by a four-electron pathway, based on the electrode materials and pH as shown below

$$\begin{array}{*{20}l} {} \hfill & {{\text{E}}_{0} } \hfill \\ {{\text{O}}_{2} + 2{\text{H}}_{2} {\text{O}} + 4{\text{e}} \to {\text{4OH}}^{ - } } \hfill & {0.41\;{\text{V}}} \hfill \\ {{\text{O}}_{2} + 2{\text{H}}_{2} {\text{O}} + 2{\text{e}} \to {\text{HO}}_{2}^{ - } + {\text{OH}}^{ - } } \hfill & { - 0.065\;{\text{V}}} \hfill \\ {{\text{HO}}_{2}^{ - } + {\text{H}}_{2} {\text{O}} + 2{\text{e}} \to 3{\text{OH}}^{ - } } \hfill & {0.867\;{\text{V}}} \hfill \\ \end{array}$$

In N-doped GQDs, the four-electron pathway is preferred for ORR, as confirmed by RDE studies and DFT calculations^10,13^. Mechanism of ORR in alkaline media on GQDs has been investigated by several groups and a clear picture has emerged suggesting two different modes of oxygen adsorption configuration, namely Yeager and Pauling configurations. DFT calculations clearly show that O_2_ gets adsorbed in Pauling mode on the surface of N-doped^34^ and N,S co-doped GQDs^35^. When we have both N and S as co-dopants, bridged adsorption configurations are more entropically favoured and this is illustrated in the thermodynamically relevant open circuit values in Table 1. However, these benefits are dominated more by kinetic effects as reflected by the higher the exchange current density and Tafel slope values.

**Table 1:** Comparison of the kinetic parameters from Tafel measurements of the GQDs; similar values for bench-marks electrocatalysts are also indicated in O_2_ saturated 0.1 M KOH. Exchange current densities were calculated from the intercepts and apparent Tafel slopes.

Catalyst	Tafel slope (mV/dec.)	Transfer coefficient, α	Exchange current density (j_0,_ A/cm^2^)	Open circuit potential (mV)
Pt	160^10^	–	3 × 10^–8^	–
Fe–N–C	120^39^	–	6.06 × 10^–6^^41^	–
Co–N–C	83^40^	–	7.07 × 10^–6^	–
Pristine GQD	80	0.7	1 × 10^–8^	123
N	100	0.51	1.1 × 10^–6^	100
N,S	120	0.49	1 × 10^–6^	75
S	90	0.62	1 × 10^–7^	230

The difference in the adsorption configuration in terms of unique structures could contribute in enhancing the performance of N-doped GQDs as compared to that of N, S-doped GQDs. For many of these 2D materials, along with doping, surface states also play a key role depending on this unique adsorption configuration as electronegativity difference can cause localized charge redistribution. However, it is difficult to separate the role of unique structures as the main involvement is through surface states.

**Figure 8:**
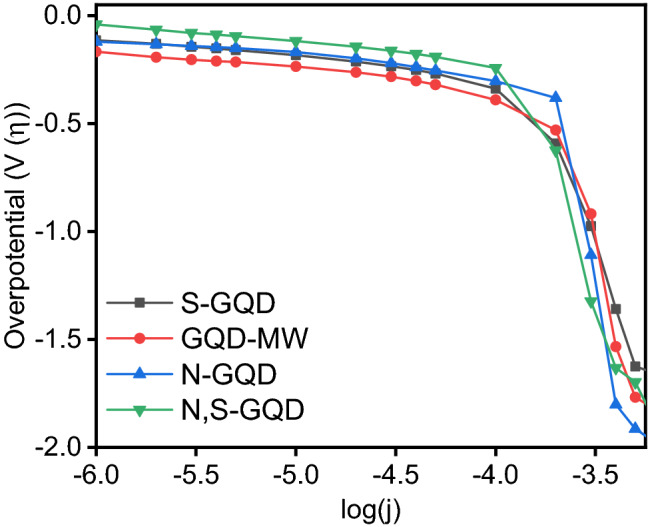
Comparison of the Tafel plots of the GQDs synthesized. Working electrode: Glassy Carbon, Counter Electrode: Pt Coil, Reference electrode: Hg/HgO. Steady-state measurements were taken in a galvanostatic manner, constant current was passed through the electrode until a steady state was reached.

Steady-state Galvanostatic polarization measurements were carried out in order to corroborate the results of the Voltammetry to calculate the apparent Tafel slopes and exchange current densities as displayed in Fig. 8. The parameters derived from the Tafel slopes are given in the Table 1, and for comparison purposes, the Tafel slopes of standard bench-mark catalysts are given. Although the values of exchange current density of N-GQDs and N,S-GQDs are very close the Tafel slope change reflects the difference in the conductivity values perhaps suggesting the role of charge redistribution around the dopant hetero-atom*.*

Doping leads to an increase in exchange current densities and the order of magnitude increase can be seen in N-GQDs, followed by N,S-GQDs and S-GQDs respectively, showing ORR is kinetically most preferable in the case of N-doped GQDs. There could be several reasons for the higher electrochemical performance of N-doped GQDs as manifested by parameters such as lower onset potential and higher exchange current density compared to those of S-doped and N, S-doped primarily due to lower activation overpotential involved in bond breaking subsequent to the Pauling adsorption configuration*.* This is also in agreement with an enhancement in conductivity values post doping, as shown by the works of other groups^36–38^.

From the above fluorescence data, voltammograms, and exchange current densities, we can see that doping causes significant changes in the GQDs and their ORR performance with profound implications for applications such as energy storage. The mechanism of ORR seems to be similar for all the GQDs considered. Perhaps, the excitation-dependent luminescent profile of N,S co-doped GQDs could be leveraged to design “smart electrocatalysts” which can either shift or in quench the luminescence when surface degradation occurs during sustained utilization but further experiments are planned on durability and degradation studies (S-03) to confirm this enticing possibility. These studies clearly indicate the importance of hetero-atom doped metal-free GQDs as a possible replacement of precious metal electrocatalysts since cost reduction and efficiency improvement are possible after establishing their durability and robustness.

## Conclusions

The ORR performance of three different types of GQDs prepared using similar methods has been compared, against their pristine counterparts to demonstrate the importance of heteroatom doping. Cyclic voltammetry undoubtedly shows a clear lowering of the onset potential with the N-doped GQDs, along with an increase in the exchange current density. The enticing possibility of connecting light emitting nature of GQDs with degradation and durability has profound implications in designing smart electrocatalysts for metal-air batteries, fuel cells and other such devices.

## Supplementary Information

Supplementary Information.

## Data Availability

All data generated or analyzed during this study are included in this published article and its supplementary information files.

## References

[1] Hoare JP (1969). The electrochemistry of oxygen. J. Electrochem. Soc..

[2] Babcock GT (1999). How Oxygen is activated and reduced in respiration. Proc. Natl. Acad. Sci. U. S. A..

[3] Damjanovic A, Genshaw MA, Bockris JO (1966). The mechanism of oxygen reduction at platinum in alkaline solutions with special reference to H_2_O_2_. J. Chem. Phys..

[4] Sasaki K, Kuttiyiel KA, Su D, Adzic RR (2011). Platinum monolayer on Ir–Fe core-shell nanoparticle electrocatalysts for the oxygen reduction reaction. Electrocatalysis.

[5] Wu G, More KL, Johnston CM, Zelenay P (2011). High-performance electrocatalysts for oxygen reduction derived from polyaniline, iron and cobalt. Science.

[6] A. D. Güçlü, P. Potasz, M. K. & Hawrylak, P. In: *Graphene Quantum Dots* (Springer Berlin Heidelberg, n.d.).

[7] Chen Y-X, Lu D, Wang G-G, Huangfu J, Wu Q-B, Wang X-F, Liu L-F, Ye D-M, Yan B, Han J (2020). Highly efficient, orange emissive graphene quantum dots prepared by acid-free method for White LEDs. ACS Sustain. Chem. Eng..

[8] Xia C, Qiu Y, Xia Y, Zhu P, King G, Zhang X, Wu Z, Kim JYT, Cullen DA, Zheng D, Li P, Shakouri M, Heredia E, Cui P, Alshareef HN, Hu Y, Wang H (2021). General synthesis of single -atom catalysts with high metal loading using graphene quantum Dots. Nat. Chem..

[9] Kalkal A, Kadian S, Pradhan R, Manik G, Packirisamy G (2021). Recent advanced in graphene quatum dot- based optical and electrochemical (bio)analytical sensors. Mater. Adv..

[10] Weber MF, Dignam MJ, Park S, Venter RD (1986). Kinetics of oxygen reduction on sputtered platinum. J. Electrochem. Soc..

[11] Jeon I-Y, Zhang S, Zhang L, Choi H-J, Seo J-M, Xia Z, Dai L, Baek J-B (2013). Edge-selectively sulfurized Graphene nanoplatelets as efficient metal-free electrocatalysts for oxygen reduction reaction: the electron spin effects. Adv. Mater..

[12] Liang J, Jiao Y, Jaroniec M, Qiao SZ (2012). Sulfur and nitrogen dual-doped mesoporous graphene electrocatalyst for oxygen reduction with synergistically enhanced performance. Angew. Chem. Int. Ed Engl..

[13] Shinde DB, Vishal VM, Kurungot S, Pillai VK (2015). Electrochemical preparation of Nitrogen-doped Graphene quantum dots and theie size-dependent electrocatalytic activity for oxygen reduction. Bull. Mater. Sci..

[14] Subramanian NP, Li X, Nallathambi V, Kumaraguru SP, Colon-Mercado H, Wu G, Lee J-W, Popov BN (2009). Nitrogen-modified carbon-based catalysts for oxygen reduction reaction in polymer electrolyte membrane fuel cells. J. Power Sources.

[15] Matter PH, Zhang L, Ozkan US (2006). The Role of nanostructure in nitrogen-containing carbon catalysts for the oxygen reduction reaction. J. Catal..

[16] Takeyasu K, Furukawa M, Shimoyama Y, Singh SK, Nakamura J (2021). Role of pyridinic nitrogen in the mechanism of the oxygen reduction reaction on carbon electrocatalysts. Angew. Chem. Int. Ed Engl..

[17] Wang X-R, Liu J-Y, Liu Z-W, Wang W-C, Luo J, Han X-P, Xi-Wen D, Qiao S-Z, Yang J (2018). Identifying the key role of pyridinic-N–Co bonding in synergistic electrocatalysis for reversible ORR/OER. Adv. Mater..

[18] Guo Q, Feng J, Liu H, Xia C, Dong H, Sun Q, Liyan Yu, Dong L (2022). Effects of hydronium and hydroxide ion/group on oxygen reduction reaction electrocatalytic activities of N-doped graphene quantum dots. Mol. Catal..

[19] Kundu S, Yadav RM, Narayanan TN, Shelke MV, Vajtai R, Ajayan PM, Pillai VK (2015). Synthesis of N F and S co-doped graphene quantum dots. Nanoscale.

[20] Kharangarh PR, Umapathy S, Singh G (2017). Effect of defects on quantum yield in blue emitting photoluminescent graphene quantum dots. J. Appl. Phys..

[21] Boonta W, Talodthaisong C, Sattayaporn S, Chaicham C, Chaicham A, Sahasithiwat S, Kangkaew L, Kulchat S (2020). The synthesis of nitrogen and sulfur co-doped graphene quantum dots for fluorescent detection of Cobalt(III) ions in water. Mater. Chem. Front..

[22] Li S, Li Y, Cao J, Zhu J, Fan L, Li X (2014). Sulfur-doped graphene quantum dots as a novel fluorescent probe for highly selective and sensitive detection of Fe ^3+^. Anal. Chem..

[23] Van Khai T, Na HG, Kwak DS, Kwon YJ, Ham H, Shim KB, Kim HW (2012). Significant enhancement of blue emission and electrical conductivity of N-doped graphene. J. Mater. Chem..

[24] Yao Y, Guo Y, Du W, Tong X, Zhang X (2018). In situ synthesis of sulfur doped graphene quantum dots decorated carbon nanoparticles as hybrid metal-free electrocatalysts for oxygen reduction reaction. J. Mater. Sci. Mater. Electron..

[25] Peng J, Gao W, Gupta BK, Liu Z, Romero-Aburto R, Ge L, Song L, Alemany LB, Zhan X, Gao G, Vithayathil SA, Kaipparettu BA, Marti AA, Hayashi T, Zhu J-J, Ajayan PM (2012). Graphene quantum dots derived from carbon fibers. Nano Lett..

[26] Permatasari FA, Aimon AH, Iskandar F, Ogi T, Okuyama K (2016). Role of C-N configurations in the photoluminescence of graphene quantum dots synthesized by a hydrothermal route. Sci. Rep..

[27] Qu D, Zheng M, Du P, Zhou Y, Zhang L, Li D, Tan H, Zhao Z, Xie Z, Sun Z (2013). Highly luminescent, sulfur, nitrogen co-doped graphene quantum dots with broad visible absorption bands for visible photocatalysts. Nanoscale.

[28] Tajik S, Dourandish Z, Zhang K, Beitollahi H, Van Le Q, Jang HW, Shokouhimehr M (2020). Carbon and graphene quantum dots: a review on synthesis, characterization, biological and sensing applications for neurotransmitter determination. RSC Adv..

[29] Li Y, Zhao Y, Cheng H, Yue Hu, Shi G, Dai L, Liangti Qu (2012). Nitrogen-doped quantum dots with oxygen-rich functional groups. J. Am. Chem. Soc..

[30] Ozonder S, Unlu C, Guleryuz C, Trabzon L (2023). Doped graphene quantum dots UV–Vis absorption spectrum: a high-throughput TDDFT study. ACS Omega.

[31] Li Q, Zhang S, Dai L, Li L-S (2012). Nitrogen-doped colloidal graphene quantum dots and their size dependent electrocatalytic activity for the oxygen reduction reaction. J. Am. Chem. Soc..

[32] Hasan MT, Gonzalez-Rodriguez R, Ryan C, Faerber N, Coffer JL, Naumov AV (2018). Photo-and electroluminescence from nitrogen-doped and nitrogen-sulfur codoped graphene quantum dots. Adv. Funct. Mater..

[33] Zhang B-X, Gao H, Li X-L (2014). Synthesis and optical properties of nitrogen and sulfur co-doped graphene quantum dots. New J. Chem..

[34] Saidi WA (2013). Oxygen reduction electrocatalysis using N -doped graphene quantum dots. J. Phys. Chem. Lett..

[35] Guo Q, Feng J, Chen D, Song N, Dong H, Yu L, Dong L (2021). Theoretical insights into enhanced electrocatalytic activity of oxygen reduction reactions on N/S- co-doped graphene quantum dots. J. Phys. Chem. C.

[36] Donghe D, Li P, Quyang J (2015). Nitrogen-doped reduced graphene oxide prepared by simultaneous thermal reduction and nitrogen doping of graphene oxide in air and its application as an electrocatalyst. ACS Appl. Mater. Interface.

[37] Tian Z, Li J, Zhu G, Lu J, Wang Y, Shi Z, Xu C (2016). Facile synthesis of highly conductive sulfur-doped reduced graphene oxide sheets Phys. Chem. Chem. Phys..

[38] Ma X, Ning G, Sun Y, Pu Y, Gao J (2014). High capacity lithium storage in nitrogen and sulfur dual doped graphene networks. Carbon.

[39] Zúñiga C, Candia-Onfray C, Venegas R, Muñoz K, Urra J, Sánchez-Arenillas M, Marco JF, Zagal JH, Recio FJ (2019). Elucidating the mechanism of the oxygen reduction reaction for pyrolyzed Fe–N–C catalysts in basic media. Electrochem. Commun..

[40] Huang D, Luo Y, Li S, Zhang B, Shen Y, Wang M (2014). Active catalysts based on Cobalt oxide @cobalt NC nanocomposites for oxygen reduction reaction in alkaline solutions. Nano Res..

[41] Lin L, Zhu Q, Xu A-W (2014). Noble-metal-free Fe–N/C catalyst for highly efficient oxygen reduction reaction under both alkaline and acidic conditions. J. Am. Chem. Soc..

