# Supplementary material for: Effect of hetero-atom doping on the electrocatalytic properties of graphene quantum dots for oxygen reduction reaction
Source: Sci Rep. 2023 Mar 30;13:5182. doi: 10.1038/s41598-023-31854-8 (PMC10063621; doi:10.1038/s41598-023-31854-8)
Supplement: Supplementary file 1 — Supplementary Information. [file 41598_2023_31854_MOESM1_ESM.docx]

Manuscript entitled “Effect of Hetero-atom Doping on the Electrocatalytic Properties of Graphene Quantum Dots for Oxygen Reduction Reaction” by Mrigaraj Goswami, Sneha Mandal, and Vijayamohanan K. Pillai

**Supplementary Information**

**S-1:**

**Synthesis of Graphene Oxide:** Graphene Oxide was synthesized using a modified Hummer’s method^[1]^. 10 gms of Graphite powder was mixed with 7.5 gm of Sodium Nitrate and were suspended in 600 ml of concentrated Sulfuric Acid, in an ice-cooled 5-litre beaker. 40 gms of Potassium Permanganate was dissolved in the acidic graphite suspension. Then, the suspension was stirred for 2 hours, at 0-4 C. Post stirring for 2 hours, the mixture was stirred at room temperature for 5 days vigorously. After that, 1 litre of 5 wt% Sulfuric acid was added slowly to the suspension, at 98 C with stirring. After the addition of the acid was completed, the resultant suspension was brought down to 60 C, and 30 ml of Hydrogen peroxide (30 wt%) over a period of 2 hours, and the temperature was brought .to room temperature from 60 C. The suspension was then put in a centrifuge at 9000 RPM, the supernatant was discarded, and then the precipitate was washed with water, by sonicating it for 30 minutes. Then, it was again put in the centrifuge, and the water was discarded. The cycle of centrifuging and washing with water was repeated 15 times. After the final wash, the resultant Graphene Oxide was dried in an air oven. Verification was done by powder XRD, as seen in Figure S-1. The amorphous nature could be attributed to the GO having few layers.

Figure S-1: Powder XRD of GO synthesized. Measurement was taken from 5^0^-90^0^ at a scan rate of 1^0^ per minute.

**S-2:**

**Synthesis of Pristine GQDs^[2,3]^**: 10 ml of Nitric acid and 30 ml of Sulphuric acid were mixed together, in that, we put 75 mg of Graphene Oxide, synthesized by modified Hummer’s method. The Graphene Oxide was suspended in the acidic mixture by sonicating it for 2 hours. After that, it was put in a microwave vessel and it was microwaved at a temperature of 120 C for 1 hour, at a power of 350 watts. After cooling the product down to room temperature, the acidic mixture was neutralised with a solution of Sodium Carbonate. Post neutralisation, the suspension was filtered through a 0.22 um PVDF filter to remove the unreacted Graphene Oxide flakes. For the purification of the quantum dots, the solution was put inside a dialysis bag (Molecular weight cut-off 3500Da), and it was dialysed for 3 days. The GQDs were collected in solid form by the process of freeze-drying.

Figure S-2: Synthesis scheme of Pristine GQDs synthesized using microwave-assisted acidic exfoliation of GO

**N-GQDs synthesis^[4]^:** The synthesis was done in a bottom-up fashion, where Citric Acid was used as the Carbon source and Urea was used as the source of both Carbon and Nitrogen, as per the method described by Qu. et. al. In 25 ml of Distilled water, 960 mg of Citric Acid (0.2 M) and 900 mg of Urea (0.6 M) were dissolved. The solution was kept in a Teflon lined stainless-steel autoclave at a temperature of 160 C for four hours. After cooling down, the solution was filtered through a 0.2 um PVDF syringe filter and the filtrate was collected. The filtrate was put inside a dialysis bag (MW cut-off 3500 Da) and was dialysed for 3 days, the water outside the dialysis bag was changed after every 6 hours. The dialysed GQD solution was used for fluorescence and UV-vis spectroscopic measurements, and it was freeze-dried to remove water and collect the N-doped GQDs.

**S-GQDs synthesis^[5]^:** S-doped GQDs were synthesized using Polyphenylene-sulfide(PPS) as the raw material using a bottom-up approach.

**PPS Synthesis: .** 7.5 gm of p-Dichlorobenzene, 2 gm of Sodium Sulfide were dispersed in N-Methyl Pyrrolidone (NMP) by stirring it vigorously for 12 hours. After a uniform suspension was obtained, it was kept inside a Teflon-lined stainless-steel autoclave at a temperature of 195 C for 12 hours. After cooling it down to room temperature, the suspension was centrifuged with methanol at 4000 RPM for 30 minutes. After discarding away the supernatant, the obtained white powder was oven-dried at 60 C overnight.

To synthesize the S-GQDs, 500 mg of the PPS synthesized earlier was dispersed in 10 ml of NMP by sonication. The suspension was kept inside a Teflon-lined stainless steel autoclave at a temperature of 290 C for 4 hours. The resultant residue was obtained by centrifugation, then the residue was oven-dried overnight. The obtained powder were the S-GQDs.

**N,S co-doped GQD synthesis^[6]:^** The synthesis was done in a bottom-up fashion, where Citric Acid was used as the Carbon source and Thiourea was used as the source of both Carbon, Sulphur, and Nitrogen, as per the method described by Qu. et. al. In 25 ml of Distilled water, 960 mg of Citric Acid (0.2 M) and 1120 mg of thiourea (0.6 M) was dissolved. The solution was kept in a Teflon-lined stainless-steel autoclave at a temperature of 160 C for four hours. After cooling down, the solution was filtered through a 0.2 um PVDF syringe filter and the filtrate was collected. The filtrate was put inside a dialysis bag (MW cut-off 3500 Da) and was dialysed for 3 days, the water outside the dialysis bag was changed after every 6 hours.

**S-3**

Preliminary durability studies carried out by using repeated polarization of N-Doped GQDs suggested very little change in half-wave potential(20 mV) but these results have been obtained only for N doped GQDs as a representative sample..


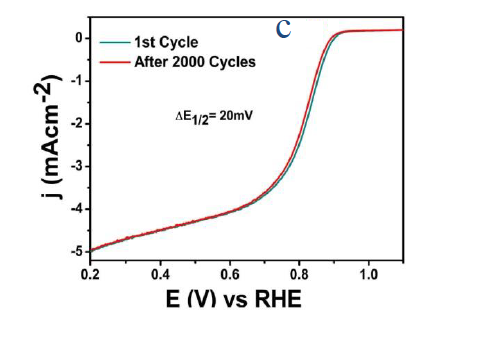


**References:**

1. Shahriary, L., & Athawale, A. A. (2014), Int. J. Renew. Energy Environ. Eng, **2**(01), 58-63.
2. J. Peng, W. Gao, B. K. Gupta, Z. Liu, R. Romero-Aburto, L. Ge, L. Song, L. B. Alemany, X. Zhan, G. Gao, S. A. Vithayathil, B. A. Kaipparettu, A. A. Marti, T. Hayashi, J.-J. Zhu, and P. M. Ajayan, Nano Lett. **12**, 844 (2012)..
3. L.-L. Li, J. Ji, R. Fei, C.-Z. Wang, Q. Lu, J.-R. Zhang, L.-P. Jiang, and J.-J. Zhu, Advanced Functional Materials **22**, 2971 (2012).
4. F. A. Permatasari, A. H. Aimon, F. Iskandar, T. Ogi, and K. Okuyama, Sci. Rep. **6**, 1 (2016).
5. S. Radhakrishnan, C. R. Rajan, and V. M. Nadkarni, J. Mater. Sci. **21**, 597 (1986).
6. D. Qu, M. Zheng, P. Du, Y. Zhou, L. Zhang, D. Li, H. Tan, Z. Zhao, Z. Xie, and Z. Sun, Nanoscale **5**, 12272 (2013).
